# Early Outcomes of Lecanemab for Alzheimer’s Disease in the Veterans Health Administration

**DOI:** 10.3390/jcm14238277

**Published:** 2025-11-21

**Authors:** Alison J. O’Donnell, Xinhua Zhao, Alyssa Parr, Sherrie Aspinall, Timothy S. Anderson

**Affiliations:** 1 Technology Enhancing Cognition and Health-Geriatric Research, Education, and Clinical Center (TECH-GRECC), VA Pittsburgh Healthcare System, Pittsburgh, PA 15240, USA; 2 Division of Geriatrics, Department of Medicine, University of Pittsburgh, Pittsburgh, PA 15213, USA; 3 Center for Health Equity Research and Promotion (CHERP), VA Pittsburgh Healthcare System, Pittsburgh, PA 15240, USA; 4 VA Center for Medication Safety, Washington, DC 20420, USA; 5 Department of Pharmacy and Therapeutics, School of Pharmacy, University of Pittsburgh, Pittsburgh, PA 15261, USA; 6 Division of General Internal Medicine, Department of Medicine, University of Pittsburgh, Pittsburgh, PA 15213, USA

**Keywords:** lecanemab, Alzheimer’s disease, implementation

## Abstract

**Background/Objectives**: While lecanemab (Leqembi) and several amyloid targeting therapies were approved for Alzheimer’s disease, questions remain on real-world implementation, safety, and effectiveness. The objective of this study was to describe the uptake and early outcomes of Veterans initiating lecanemab. **Methods**: This retrospective cohort study included Veterans who initiated lecanemab in the Veteran’s Health Administration (VHA) between October 2023 and July 2024. Treatment persistence and monitoring, change in Montreal Cognitive Assessment (MoCA) score, incidence of adverse events, including amyloid-related imaging abnormalities (ARIA), and healthcare utilization were analyzed at 7 months. **Results**: Overall, 32 Veterans (mean [SD] age 75.3 [6.0] years, 100% male, 97% white, 84% urban dwelling) initiated lecanemab. Seventeen patients (53%) had mild cognitive impairment, 15 (47%) had mild dementia; mean baseline MoCA score was 21.3 (SD 3.4). At 7 months following treatment initiation, we assessed process, safety, and effectiveness outcomes. Process outcomes: In all, 25 patients (78%) were persistent with treatment. Safety outcomes: Three patients (9%) experienced a stroke, and 7 (22%) experienced ARIA. Effectiveness outcomes: Only 12 (38%) patients had a MoCA completed by 7 months, and the mean change in MoCA was 0.0 (SD 3.7, *p* = 1.0). A follow-up amyloid positron emission tomography (PET) scan was completed by 9 (28%) patients, and 5 had reductions in amyloid. **Conclusions**: Initial observations in a small VHA cohort suggest that uptake of lecanemab was limited, and the finding that nearly 30% of patients experienced ARIA or stroke within 7 months of initiation underscores the importance of monitoring the lecanemab safety and effectiveness long-term. These early findings should be interpreted cautiously given the limited sample size and very limited follow-up MoCA data.

## 1. Introduction

Alzheimer’s dementia affects an estimated 7.2 million Americans age 65 and older, and approximately 1 in 9 people (11%) age 65 and older have Alzheimer’s dementia [1]. Alzheimer’s disease (AD) disproportionally impacts the Veteran population. An estimated 450,000 Veterans live with AD, and the number of Veterans living with dementia will increase to nearly 340,000 by 2030 with many more living with mild cognitive impairment [2]. The number of Veterans with dementia is expected to grow as the Veteran population ages because the incidence of dementia increases with older age. Dementia places a heavy emotional, financial, and physical burden on patients, families, and the healthcare system. Additionally, dementia contributes to unnecessary emergency department visits, hospitalizations, and institutionalization. The cost of care for all patients with dementia in 2025 dollars is estimated at USD 384 billion, which does not include the estimated USD 413 billion in unpaid care provided by informal caregivers [1].

Veterans are at greater risk of dementia due to exposure to military-related risk factors (e.g., traumatic brain injury (TBI), post-traumatic stress disorder (PTSD), and environmental exposures) and high prevalence of non-military risk factors, such as cardiovascular disease. Studies have shown a strong link between TBI and dementia. The physical impacts of service-related injuries, such as TBI, can lead to chronic traumatic encephalopathy (CTE) and other neurodegenerative conditions, accelerating cognitive decline. Veterans who have experienced TBI often show a higher incidence of cognitive impairment as they age, highlighting the long-term repercussions of these injuries. Similarly, PTSD has been associated with an increased risk of dementia in Veterans. Chronic stress and associated biological changes that occur with PTSD may lead to pathways that contribute to cognitive impairment. Veterans with PTSD may exhibit higher levels of stress hormones, which can impact brain function over time and increase the risk of developing dementia. Moreover, environmental exposures during military service, such as exposure to burn pits and certain chemicals, have raised concerns about long-term impacts on brain health. In addition to military-specific factors, Veterans often exhibit a high prevalence of non-military risk factors that contribute to dementia. Cardiovascular disease is a known risk factor for vascular dementia and other forms of cognitive decline. Veterans often possess a higher risk of cardiovascular disease due to stress, lifestyle factors, and other comorbid conditions. Hypertension, hyperlipidemia, and uncontrolled diabetes are also prevalent among Veterans, which may be associated with accelerated cognitive decline due to impacts on blood flow, atherosclerotic plaques, and glucose metabolism in the brain, respectively [3,4].

Health disparities further exacerbate the issue among Veterans, particularly Black and Hispanic Veterans, who face higher rates of dementia compared to their White counterparts. Socioeconomic challenges such as poverty, lack of access to quality healthcare, and educational disparities amplify these risks. Low socioeconomic status can affect diet, access to medical care, and overall health behaviors, thereby increasing the risk of cognitive decline. Veterans residing in disadvantaged neighborhoods often experience compounded effects due to reduced access to medical resources and support services, further increasing their vulnerability to cognitive decline. These areas may lack the necessary healthcare infrastructure to provide early diagnosis and ongoing management of dementia, leading to worse outcomes for affected individuals [3,4].

Historically, there have been few disease-modifying therapies for AD, the most common cause of dementia. Cholinesterase inhibitors and memantine may provide symptomatic treatment for AD, but do not alter the underlying disease trajectory. From 2003 to 2021, there were no new approved treatments for AD. Recently, several recombinant monoclonal antibodies directed against amyloid beta have been studied in large clinical trials, leading to new hope for disease modification. Lecanemab was the first of these treatments to receive full Food & Drug Administration (FDA) approval and to become available in Veterans Health Administration (VHA).

The Clarity AD trial, a large, phase 3 randomized trial of lecanemab in patients with early AD, demonstrated that lecanemab modestly slows decline on measures of cognition and function by approximately 27% after 18 months of treatment compared to placebo. While the trial did not show a stoppage or reversal of decline, this marked a significant breakthrough in AD treatment, offering a potential means to modify disease progression rather than merely addressing symptoms. However, treatment with lecanemab is not without risks. Lecanemab was associated with infusion-related reactions in 26.4% of participants and amyloid-related imaging abnormalities (ARIA) in 21.5%, with most ARIA events occurring early within the first 1 to 2 months of treatment. The rates of ARIA with edema or effusions (ARIA-E) and ARIA with cerebral microhemorrhages, cerebral macrohemorrhages, or superficial siderosis (ARIA-H) in the treatment group were 12.5% for ARIA-E and 17.3% for ARIA-H, and 8.2% of patients experienced both ARIA-H and ARIA-E. While most ARIA events were asymptomatic—with symptomatic ARIA-E occurring in 2.8% of intervention participants and symptomatic ARIA-H occurring in about 0.7%—ARIA can be severe and even result in death. Notably, the risk of ARIA-E and ARIA-H was markedly higher in patients homozygous for the Apolipoprotein E (APOE) ε4 allele [5].

In addition to the concerns regarding side effects, the evaluation process for lecanemab is extensive and involves extensive clinical evaluations, laboratory tests, APOE genetic testing, and advanced imaging, including magnetic resonance imaging (MRI) brain and amyloid positron emission tomography (PET) scans to determine eligibility. Furthermore, treatment with lecanemab may be burdensome to patients as it involves intravenous (IV) administration of the medication every two weeks and frequent monitoring with clinical assessments and neuroimaging to ensure safety [5].

These challenges underscore the substantial infrastructure needed to administer lecanemab safely, which includes acquiring necessary personnel, expertise, and resources such as advanced imaging. As a result of these concerns, the VA Center for Medication Safety (VAMedSAFE) developed criteria for use and a real-time medication use evaluation (i.e., registry) to monitor for early safety signals. The medication use evaluation follows the care of patients on lecanemab to ensure that sites have infrastructure in place, meet appropriate criteria for use, complete monitoring protocols, and report and address adverse drug events. According to VAMedSAFE, the criteria for use are recommendations grounded in medical evidence, clinician feedback, and expert opinion. They are designed to aid practitioners in making clinical decisions, standardize and enhance the quality of patient care, and encourage cost-effective prescribing of medications. The recommendations reflect findings from the trials and real-world practices based on recommendations from clinicians and other experts, including the use of the Montreal Cognitive Assessment (MoCA) and other similar instruments over the Clinical Dementia Rating-Sum of Boxes (CDR-SB) scale [6]. The MoCA is considered the standard at the VHA, among neurologists, geriatric psychiatrists, and geriatricians prescribing lecanemab. It is strongly correlated with the CDR-SB, and crosswalks have been developed between CDR-SB and MoCA [7].

As trials of these therapies often excluded patients with comorbidities such as prior stroke and psychiatric conditions, questions remain on real-world safety and effectiveness. Additionally, racial and ethnic disparities in clinical trial participation raise questions about the generalizability of results to groups that were not well represented in the trials as well as concerns about access to these novel treatment options for these populations [5]. Despite promising clinical trial results, uncertainties remain regarding the real-world implementation of lecanemab in the VHA, particularly its safety and effectiveness among Veterans with complex comorbidities and military-related risk factors not adequately represented in trials. Evaluating initial uptake and outcomes in the small VHA population receiving lecanemab is important both to evaluate outcomes in populations not represented in the trials and ensure that Veterans have equal access to these treatments, regardless of race or ethnicity. Given these research gaps, the objective of this study was to evaluate the initial uptake and outcomes of Veterans initiating lecanemab in the VHA.

## 2. Materials and Methods

### 2.1. Study Design and Population

This retrospective cohort study included all Veterans across the national VHA initiating lecanemab following approval in the VHA between October 2023 and July 2024. A 7-month data extraction period was used following initiation of lecanemab and until study end (28 February 2025). Data sources included VHA Corporate Data Warehouse (CDW) data and chart review conducted independently by two clinicians (AJO, TSA), a geriatrician and a general internist. Clinicians were not blinded; however, study outcomes were predetermined and exact wording was extracted from clinician and radiology notes to reduce to potential for bias. Additionally, chart reviews were conducted independently, and data were discussed at a weekly team meeting to confirm accuracy of data extraction. This study involved a comprehensive search of the entire national VHA system.

### 2.2. Covariates

Baseline data were extracted on demographics, MRI brain imaging, amyloid PET, medication use, dementia diagnosis/staging, and APOE genetic testing. Chart review of lecanemab evaluation clinic notes was used to collect dementia diagnosis, MoCA score [8], Functional Assessment Screening Tool (FAST) stage [9], and APOE genotype.

### 2.3. Outcomes

We examined process, effectiveness, and safety outcomes. Process measures extracted from CDW included doses of lecanemab received, monitoring MRI brain completion, and treatment discontinuation (defined as >90 days without an infusion and selected after careful consideration of treatment patterns with recognition of the limitations to any operation definition). Effectiveness was measured by change in MoCA score and amyloid plaque burden on amyloid PET. Reduction in amyloid was determined based on the radiology report. Amyloid PET scans were rated visually. Safety outcomes included death, adverse events (ARIA, stroke, infusion reactions, falls), and acute care utilization. ARIA and stroke were extracted from monitoring brain MRIs using the same definitions as in the Clarity AD trial [5] with the understanding that this may underestimate subclinical events. ARIA severity and sub-type (i.e., ARIA-E and/or ARIA-H) were extracted from radiology reports [10]. Acute care utilization included all hospitalizations and emergency department or urgent care visits.

### 2.4. Statistical Analysis

In the primary analysis, all outcomes were ascertained at 7 months. A 7-month period was chosen given VHA requirements for 6-month follow-up cognitive testing and brain MRIs prior to the 5th, 7th, and 14th infusions. Descriptive statistics were used to summarize baseline characteristics and frequencies of most outcomes due to the small sample size. A Wilcoxon signed-rank test was used to compare baseline and follow-up MoCA scores. In secondary analyses, we examined outcomes until study end (28 February 2025). Statistical analyses were conducted using SAS 8.3. (version 8.3, Cary, NC, USA)

### 2.5. Data Availability Statement

The data in this study are not publicly available due to institutional policies. Access to data can only be granted upon appropriate request and approval by the IRB. We will share code lists and variable definitions upon reasonable request.

### 2.6. Standard Protocol Approvals

Ethical review and approval were waived for this study by the VA Pittsburgh Healthcare System Institutional Review Board (protocol code 1798962-1, initial review and exempt determination granted on 7 May 2024).

## 3. Results

### 3.1. Baseline Characteristics

Overall, 32 Veterans initiated lecanemab in the VHA between October 2023 and July 2024 across 4 of the 170 VHA medical centers. The mean age was 75.3 (SD 6.0) and all 32 patients (100%) were male. Almost all (*n* = 31, 97%) were white and identified as not Hispanic or Latino (*n* = 30, 94%). Most (*n* = 27, 84%) were urban dwelling and resided in the Northeast region of the United States (*n* = 7, 84%) (Table 1).

Of the 32 patients, 17 (53%) had mild cognitive impairment and 15 (47%) had mild dementia. Mean baseline MoCA score on a scale of 0 to 30 was 21.3 (SD 3.4). One patient had a FAST stage of 2, 15 (47%) had a FAST stage of 3, and 16 (50%) had a FAST stage of 4. Half were heterozygous for APOE ε4, half had no copies of the APOE ε4 allele, and none were homozygous. Three patients were taking low-dose aspirin at baseline. No patients were on anticoagulants.

Nearly all patients (*n* = 31, 97%) had an MRI brain within 180 days of their first infusion. Six patients had a baseline MRI brain with a prior stroke, and nearly half (*n* = 15, 48%) had microhemorrhages present. All patients with baseline microhemorrhages had less than 10 present, in line with the recommended criteria for use.

A baseline amyloid PET scan was present for 29 Veterans, 1 had amyloid confirmation by cerebral spinal fluid (CSF), and 2 had no documented amyloid confirmation by CSF or amyloid PET. Of the 29 patients with a baseline amyloid PET, 14 had moderate/frequent amyloid, 6 had mild amyloid, 8 had amyloid present but not graded, and 1 had an uncertain amount of amyloid (Table 2).

### 3.2. Process, Effectiveness, and Safety Outcomes at 7 Months

At 7 months following treatment initiation, 25 (78%) patients were persistent with treatment, 3 patients had gaps between 30 and 89 days, and 4 had gaps > 90 days (Table 2). Brain MRI monitoring occurred regularly, with 29 (97%) patients receiving an MRI between the 4th and 5th infusions, 26 (93%) between the 6th and 7th infusions, and 22 (85%) between the 13th and 14th infusions.

**Figure 1:**
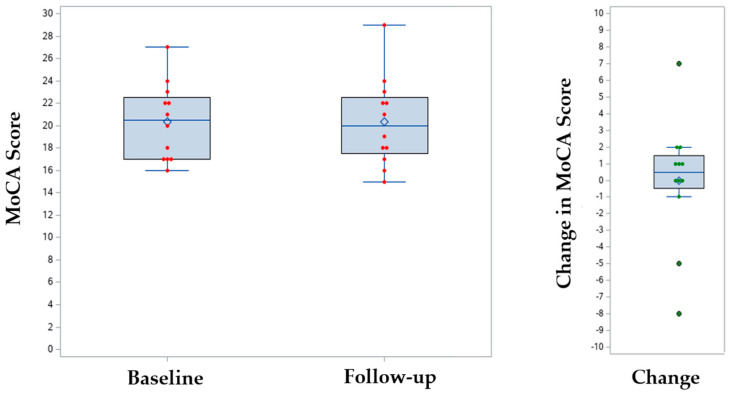
Change in Montreal Cognitive Assessment (MoCA) score in a limited sample at 7-Months following lecanemab administration (*n* = 12). The red dots reflect the raw scores and the green dots reflect the change in MoCA score. Box plot of baseline and 7-month follow-up MoCA scores. Baseline: mean = 20.3, SD = 3.4. 7-month follow-up: mean = 20.3, SD = 4.0. Wilcoxon signed-rank test: *p* = 0.96.

Only 12 patients (38%) had a documented MoCA following lecanemab initiation, and the mean change in MoCA score compared to baseline was 0.0 (SD 3.7, *p* = 0.96) (Table 2, Figure 1). Of the 20 patients (62%) with missing MoCA data, many of the scores fell outside of the predetermined follow-up window of 5 to 7 months or were not performed. Of the 9 (28%) patients with follow-up amyloid PET scans, 5 had reductions in amyloid, 2 had no change, and 2 were uncertain.

During 7-month follow-up, 3 patients experienced acute stroke based on MRI brain radiology reports (none requiring hospitalization), and 7 (22%) patients experienced ARIA. One patient experienced both a stroke and ARIA. Three patients had ARIA-E (2 mild, 1 moderate), 5 had ARIA-H (4 mild, 1 severe), and 1 had both (moderate ARIA-E/ARIA-H). None of the 3 patients on aspirin developed ARIA, and no patients were on anticoagulants. Stratifying patients with ARIA by APOE status, 5 of the 7 patients did not have any copies of the APOE ε4 allele and the other two were heterozygous for the APOE ε4 allele. The 1 patient with both ARIA-E and ARIA-H was heterozygous for the APOE ε4 allele. No patients died, 3 were hospitalized within VHA (2 for infusion reactions), and 15 (47%) had a total of 34 VHA emergency department or urgent care visits (1 for falls and 1 for infusion reaction).

### 3.3. Process, Effectiveness, and Safety Outcomes at Study Completion

The median follow-up to study completion was 323 days (range 213 to 500). At study completion, 8 patients stopped lecanemab for more than 30 days, of which 6 had gaps of >90 days. Reasons for holding or discontinuing lecanemab included adverse events (including 2 with ARIA, 1 with stroke, and 1 with weakness), amyloid clearance (*n* = 1), patient preference (*n* = 1), and unrelated illnesses (*n* = 2). No additional ARIA or stroke events occurred after 7-month follow-up to study completion (Table 3).

## 4. Discussion

In the first year lecanemab was available in VHA, the few patients initiated on treatment were mostly white, male, and urban residents, and adherence to process measures varied, with high adherence to monitoring brain MRIs although few patients received cognitive assessments. At 7 months, nearly 30% of patients experienced ARIA or stroke, and nearly one-quarter discontinued treatment or had prolonged treatment holds. One patient experienced both stroke and ARIA.

These results build on one prior study examining the initial rollout of lecanemab at a single academic specialty memory clinic, finding similar rates of ARIA and a 10% discontinuation rate [11] as well as real-world data presented at the Alzheimer’s Association International Conference in July 2025 [12]. In the Clarity AD trial, 21% of patients experienced ARIA by 18 months [5], a similar proportion observed by 7 months in the current study. While we hypothesized that lower rates of ARIA may be observed in the VHA due to stricter eligibility criteria (e.g., APOE ε4 homozygous status and anticoagulant use are contraindications to treatment), this was not observed. It is possible that the prevalence of military-related risk factors puts Veterans at risk for ARIA and stroke; however, the sample is too small to draw any conclusions. While the majority of patients with ARIA (5 of 7) did not have any copies of the APOE ε4 allele, the sample is again too small to draw any conclusions.

The rate of stroke was not reported in the published Clarity AD trial or supplements. However, the finding that 3 patients experienced stroke, one of whom also experienced ARIA, underscores the importance of continual monitoring of lecanemab safety in real-world populations.

The primary effectiveness measure for the Clarity AD trial was the CDR-SB score [5], which is less often used in clinical practice than the MoCA. While MoCA scores were unchanged at 7 months in this study, the small sample size, incomplete data, and short follow-up period limits the ability to draw any conclusions regarding effectiveness. The effectiveness of lecanemab remains controversial given the difference in CDR-SB score in the Clarity AD trial was smaller than proposed clinically meaningful differences [13].

Consistent with prior studies [5,11], 97% of the participants identified as white and 94% as not Hispanic or Latino. While we hypothesized that there would be fewer disparities within the VA given fewer cost-related barriers to care, the first sites that were able to administer lecanemab were mostly urban and associated with large, academic medical centers. This study further emphasizes the strong disparities in uptake of amyloid targeting therapies in the U.S. and the need for further investigation to ensure that Veterans have equal access to these treatments, regardless of race or ethnicity.

The VHA through VAMedSAFE has made significant efforts to monitor the safety of Veterans on these therapies, developing criteria for use and requiring a national real-time medication use evaluation to monitor the safe and appropriate use of lecanemab [6]. The low uptake of lecanemab may be at least partially attributable to VHA’s strict eligibility criteria. The observed high rates of adherence to safety monitoring requirements can likely be attributed to VAMedSAFE’s oversight of prescribing and low out-of-pocket cost to Veterans. This may suggest that reducing financial obstacles in commercial healthcare systems could improve patient compliance with regular monitoring and treatment, potentially reducing patient risk and lowering discontinuation related to cost. However, missing data—specifically follow-up MoCA scores—reflects real-world practices as well as VAMedSAFE’s role to provide recommendations and allow clinicians and local medical centers to use clinical judgment [6]. Additionally, adverse events may have been underreported and reliance on MRI radiology reports may lead to underreporting of subclinical events. As a result of these early findings, VAMedSAFE has provided feedback to sites regarding process, safety, and effectiveness measures, and has encouraged sites to improve adherence to reporting metrics.

These findings should be considered in the context of the following limitations. Most importantly, these early findings should be interpreted cautiously given the small sample size, and particularly the few patients with follow-up MoCA data, which limits the ability to draw any conclusions regarding effectiveness. Second, these findings may not be generalizable to other populations and health systems. The VHA serves a predominately male population, and patients face fewer cost-related barriers to care. Third, no comparison group was used in this pilot study. Fourth, the study design was retrospective and relied on real-world data. Fifth, limited information was collected on patients with stroke. Finally, there was a potential for reporting and misclassification bias, which we attempted to mitigate by predetermining study outcomes, relying on objective data, and having two clinicians review charts independently and discussing at a weekly team meeting to confirm accuracy of data extraction.

This paper notes early safety signals, but the small sample size limits generalization. Findings are preliminary and hypothesis-generating, not definitive. As the use of these therapies expands, these early findings indicate a need for close monitoring of real-world safety and effectiveness. Larger, multi-center prospective VHA registry studies are needed that include standardized effectiveness follow-up and are inclusive of diverse populations.

## Figures and Tables

**Table 1:** Baseline Demographics of Veterans who initiated lecanemab in the VHA between October 2023 and July 2024.

Baseline Demographics	Participants
Age, mean (SD), years	75.3 (6.0)
Birth sex, male, *n* (%)	32 (100)
Race, *n* (%)	
White	31 (97)
Declined to Answer	1 (3)
Ethnicity, *n* (%)	
Hispanic or Latino	1 (3)
Not Hispanic or Latino	30 (94)
Declined to answer	1 (3)
Region, *n* (%)	
Northeast	27 (84)
Midwest	0 (0)
South	3 (9)
West	2 (6)
Rurality, *n* (%)	
Rural	5 (16)
Urban	27 (84)

Abbreviations: SD = standard deviation.

**Table 2:** Clinical characteristics of Veterans who initiated lecanemab in the VHA between October 2023 and July 2024.

Clinical Characteristics	Participants
Alzheimer’s diagnosis, *n* (%)	
Mild cognitive impairment	17 (53)
Mild dementia	15 (47)
Functional Assessment Staging Test, *n* (%)	
Stage 2	1 (3)
Stage 3	15 (47)
Stage 4	16 (50)
Baseline MoCA score, mean (SD)	21.3 (3.4)
Apolipoprotein E genotype, *n* (%)	
Non-carrier	16 (50)
Heterozygous	16 (50)
Baseline aspirin use, *n* (%)	3 (9)
MRI brain ≤ 180 days prior to first infusion, *n* (%)	31 (97)
Brain MRI with prior stroke	6 (19)
Brain MRI with microhemorrhages (<10)	15 (48)
Amyloid confirmation, *n* (%)	
Amyloid PET scan prior to first infusion *	29 (91)
Moderate to frequent	14/29 (48)
Mild	6/29 (21)
Amyloid present, not graded	8/29 (28)
Uncertain	1/29 (3)
Amyloid-positive CSF testing, no amyloid PET documented	1 (3)
No documented CSF testing or amyloid PET	2 (6)

Abbreviations: CSF = cerebral spinal fluid; MoCA = Montreal Cognitive Assessment; MRI = magnetic resonance imaging; PET = positron emission tomography; * Extracted from radiology notes.

**Table 3:** Safety, effectiveness, and process outcomes of Veterans who initiated lecanemab in the VHA between October 2023 and July 2024.

	7-Month Follow-Up	Study Completion
Median days of follow-up, range	210	323 (213–500)
Process and Safety Outcomes		
Number of infusions, median (range)	15 (2–16)	21 (2–35)
Gap between consecutive infusions or until the end of the study period, *n* (%)		
<30 days	25/32 (78)	24/32 (75)
30–89 days	3/32 (9)	2/32 (6)
≥90 days *	4/32 (13)	6/32 (19)
MRI brain for monitoring, *n* (%) **		
Between the 4th and 5th infusion	29/30 (97)	29/30 (97)
Between the 6th and 7th infusion	26/28 (93)	26/29 (90)
Between the 13th and 14th infusion	22/26 (85)	23/29 (79)
Initial amyloid PET follow-up, *n* (%)		
Received an initial follow-up amyloid PET scan	9/32 (28)	16/32 (50)
Decreased amyloid	5/9 (56)	9/16 (56)
No change in amyloid	2/9 (22)	4/16 (25)
Uncertain	2/9 (22)	3/16 (19)
Death, *n* (%)	0	0
ARIA, *n* (%)		
Any ARIA	7/32 (22)	7/32 (22)
ARIA-E	3/32 (9)	3/32 (9)
Mild	2/32 (6)	2/32 (6)
Moderate	1/32 (3)	1/32 (3)
ARIA-H	5/32 (16)	5/32 (16)
Mild	4/32 (13)	4/32 (13)
Moderate	1/32 (3)	0/32
Severe	0/32	1/32 (3)
Both ARIA-E and ARIA-H	1/32 (3)	1/32 (3)
Any ARIA or stroke	9/32 (28)	9/32 (28)
Acute stroke	3/32 (9)	3/32 (9)
Death	0/32	0/32
All-cause hospitalization, *n* (%)	3/32 (9)	3/32 (9)
Infusion-reaction-related	2/32 (6)	2/32 (6)
Lumbar spinal stenosis	1/32 (3)	1/32 (3)
All-cause emergency department or urgent care visit		
Number of patients, *n* (%)	11/32 (34)	15/32 (47)
Total visits, *n*	21	34
Fall-related, *n* (%)	1/21 (3)	2/34 (6) ***
Infusion-reaction-related, *n* (%)	1/21 (3)	1/34 (3)
**Effectiveness Outcomes**		
Cognitive function, *n* (%)		
Any MoCA between 5 and 7 months	12/32 (38)	NA
Baseline MoCA, mean (SD)	20.3 (3.4)	NA
Follow-up MoCA between 5 and 7 months, mean (SD)	20.3 (4.0)	NA
Change in MoCA, mean (SD)	0.0 (3.7) ****	NA

Abbreviations: ARIA = amyloid-related imaging abnormalities; ARIA-E = amyloid-related imaging abnormalities with edema and/or effusion; ARIA-H = amyloid-related imaging abnormalities with hemorrhage and/or superficial siderosis; MoCA = Montreal Cognitive Assessment, range 0–30; NA = not applicable; MRI = magnetic resonance imaging; PET = positron emission tomography. * Of the 6 people with a ≥90 gap, one patient restarted lecanemab during study follow-up. ** Patients who stopped or held lecanemab prior to infusion not included in this analysis. Monitoring intervals based on VHA guidance for required MRI brain imaging frequency. *** Including 2 falls from 1 patient. **** *p* = 0.96.

## Data Availability

The data in this study are not publicly available due to institutional policies. Access to data can only be granted upon appropriate request and approval by the IRB.

## Abbreviations

The following abbreviations are used in this manuscript:

VHA	Veteran’s Health Administration
MoCA	Montreal Cognitive Assessment
ARIA	Amyloid-related imaging abnormalities
AD	Alzheimer’s disease
CDW	Corporate Data Warehouse
APOE	Apolipoprotein E
FAST	Functional Assessment Screening Tool
ARIA-E	ARIA with edema or effusions
ARIA-H	ARIA with cerebral microhemorrhages, cerebral macrohemorrhages, or superficial siderosis
CSF	Cerebral spinal fluid
MRI	Magnetic resonance imaging
PET	Positron emission tomography
CDR–SB	Clinical Dementia Rating–Sum of Boxes

## References

[1] Alzheimer’s Association (2025). 2025 Alzheimer’s Disease Facts and Figures. Alzheimer’s Dement..

[2] Statistical Projections of Alzheimer’s Dementia for VA Patients, VA Enrollees, and U.S. Veterans Fiscal Years 2021 and 2033. https://www.va.gov/GERIATRICS/docs/VHA_ALZHEIMERS_DEMENTIA_Statistical_Projections_FY21_and_FY33_sgc121820.pdf.

[3] Walker L.E., Poltavskiy E., Janak J.C., Beyer C.A., Stewart I.J., Howard J.T. (2019). US Military Service and Racial/Ethnic Differences in Cardiovascular Disease: An Analysis of the 2011–2016 Behavioral Risk Factor Surveillance System. Ethn. Dis..

[4] Yaffe K., Vittinghoff E., Lindquist K., Barnes D., Covinsky K.E., Neylan T., Kluse M., Marmar C. (2010). Posttraumatic Stress Disorder and Risk of Dementia Among US Veterans. Arch. Gen. Psychiatry.

[5] Van Dyck C.H., Swanson C.J., Aisen P., Bateman R.J., Chen C., Gee M., Kanekiyo M., Li D., Reyderman L., Cohen S. (2023). Lecanemab in Early Alzheimer’s Disease. N. Engl. J. Med..

[6] VA Formulary Advisor: LECANEMAB-IRMB INJ, SOLN. https://www.va.gov/formularyadvisor/drugs/4041980-LECANEMAB-IRMB-INJ-SOLN.

[7] Ackley S.F., Wang J., Chen R., Hill-Jarrett T.G., Rojas-Saunero L.P., Stokes A., Shah S.J., Glymour M.M., Initiative F.T.A.D.N. (2025). Methods to crosswalk between cognitive test scores using data from the Alzheimer’s Disease Neuroimaging Cohort. Alzheimer’s Dement..

[8] Nasreddine Z.S., Phillips N.A., Bédirian V., Charbonneau S., Whitehead V., Collin I., Cummings J.L., Chertkow H. (2005). The Montreal Cognitive Assessment, MoCA: A brief screening tool for mild cognitive impairment. J Am Geriatr Soc..

[9] Reisberg B., Jamil I.A., Khan S., Monteiro I., Torossian C., Ferris S., Sabbagh M., Gauthier S., Auer S., Shulman M.B., Abou-Saleh M.T., Katona C., Kumar A. (2010). Staging Dementia. Principles and Practice of Geriatric Psychiatry.

[10] Sims J.R., Zimmer J.A., Evans C.D., Lu M., Ardayfio P., Sparks J., Wessels A.M., Shcherbinin S., Wang H., Nery E.S.M. (2023). Donanemab in Early Symptomatic Alzheimer Disease: The TRAILBLAZER-ALZ 2 Randomized Clinical Trial. JAMA..

[11] Paczynski M., Hofmann A., Posey Z., Gregersen M., Rudman M., Ellington D., Aldinger M., Musiek E.S., Holtzman D.M., Bateman R.J. (2025). Lecanemab Treatment in a Specialty Memory Clinic. JAMA Neurol..

[12] Two-Year Real-World Study of LEQEMBI^®^ in the United States Presented at Alzheimer’s Association International Conference (AAIC) 2025. https://media-us.eisai.com/2025-07-30-Two-Year-Real-World-Study-of-LEQEMBI-R-in-the-United-States-Presented-at-Alzheimers-Association-International-Conference-AAIC-2025.

[13] Lansdall C.J., McDougall F., Butler L.M., Delmar P., Pross N., Qin S., McLeod L., Zhou X., Kerchner G.A., Doody R.S. (2023). Establishing Clinically Meaningful Change on Outcome Assessments Frequently Used in Trials of Mild Cognitive Impairment Due to Alzheimer’s Disease. J. Prev. Alzheimer’s Dis..

